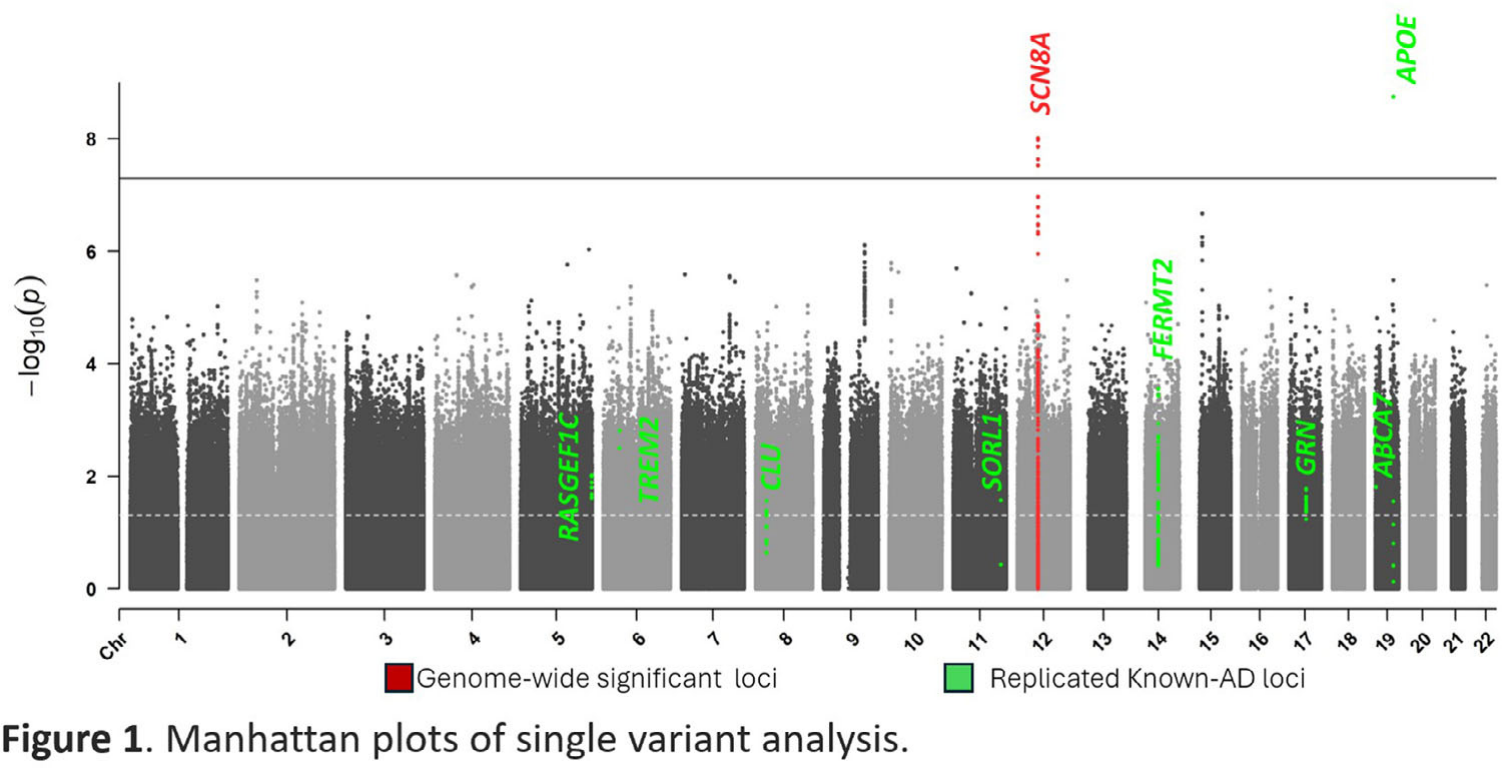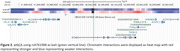# Significant association between *SCN8A* and Alzheimer disease in Puerto Ricans

**DOI:** 10.1002/alz70855_098248

**Published:** 2025-12-23

**Authors:** Bilcag Akgun, Wanying Xu, Kara L Hamilton‐Nelson, Luciana Bertholim Nasciben, Kyle M Scott, Larry D Adams, Jose J. Sanchez, Glenies S. Valladares, Concepcion Silva‐Vergara, Vanessa C. Rodriguez, Pedro R Mena, Sabrina M. Mas, Mariangelie Lopez, Katrina Celis, Patrice G Whitehead, Michael B. Prough, Anthony J Griswold, Clifton L. Dalgard, Briseida E. Feliciano‐Astacio, Giuseppe Tosto, Jonathan L Haines, William S Bush, Christiane Reitz, Brian W Kunkle, Goldie S Byrd, Rufus O Akinyemi, Adesola Ogunniyi, Susan H. Blanton, Liyong Wang, Katalina F. McInerney, Michael L Cuccaro, Fulai Jin, Jeffery M Vance, Farid Rajabli, Margaret Pericak‐Vance

**Affiliations:** ^1^ John P. Hussman Institute for Human Genomics, University of Miami Miller School of Medicine, Miami, FL, USA; ^2^ Department of Genetics and Genome Sciences, School of Medicine, Case Western Reserve University, Cleveland, OH, USA; ^3^ The Biomedical Sciences Training Program (BSTP), School of Medicine, Case Western Reserve University, Cleveland, OH, USA; ^4^ University of Miami Miller School of Medicine, Miami, FL, USA; ^5^ Dr. John T. Macdonald Foundation Department of Human Genetics, University of Miami Miller School of Medicine, Miami, FL, USA; ^6^ Department of Anatomy, Physiology, and Genetics, Uniformed Services University of the Health Sciences, Bethesda, MD, USA; ^7^ Universidad Central del Caribe, Bayamon, PR, USA; ^8^ Gertrude H. Sergievsky Center, Taub Institute for Research on the Aging Brain, Departments of Neurology, Psychiatry, and Epidemiology, College of Physicians and Surgeons, Columbia University, New York, NY, USA; ^9^ Cleveland Institute for Computational Biology, Department of Population & Quantitative Health Sciences, School of Medicine, Case Western Reserve University, Cleveland, OH, USA; ^10^ Wake Forest University School of Medicine, Winston‐Salem, NC, USA; ^11^ College of Medicine, University of Ibadan, Ibadan, Oyo, Nigeria; ^12^ Institute for Advanced Medical Research and Training, College of Medicine, University of Ibadan, Ibadan, Nigeria; ^13^ Department of Neurology, University of Miami Miller School of Medicine, Miami, FL, USA

## Abstract

**Background:**

Puerto Ricans (PR), the second‐largest Hispanic/Latino population in the continental US, are an admixed population with European (EU), African (AF), and Amerindian ancestries. Our recently published genome‐wide association study (GWAS) and admixture mapping in a PR cohort identified a suggestive Alzheimer disease (AD) risk locus on chromosome 12 (Akgun et al., 2024), overlapping a previously known linkage region (12q13) that has eluded further delineation (Pericak‐Vance et al., 1997). We expanded the PR cohort with newly recruited individuals and conducted an expanded GWAS and meta‐analysis to investigate associations further in the 12q13 region, characterize known AD loci, and identify novel AD susceptibility loci.

**Method:**

We analyzed 867 PR individuals (449 AD; 428 cognitively unimpaired), including 219 newly recruited individuals through the READD‐ADSP, and summary statistics from 648 individuals the previous publication. We imputed genotype data to the NHLBI TOPMed haplotype reference panel and performed GWAS with a generalized linear mixed model adjusting for sex, age, population substructure, and genetic relationship matrix. We meta‐analyzed the two cohorts using an inverse‐variance‐weighted fixed‐effects model. To identify target genes at top novel loci, we examined chromatin interactions using brain autopsy samples from different ancestries (Celis et al., 2023). Enhanced Hi‐C Capture Analysis (eHiCA) was performed by using a 5kb bait centered on the top marker and extracting all chromatin interactions with the bait from a +/‐ 2Mb of the surrounding genomic region.

**Result:**

We identified a genome‐wide significant (*p* <5x10^‐8^) signal (rs4761988, *p* = 9.7x10^‐9^) within the first intron of *SCN8A* on chromosome 12 (Figure 1), overlapping a significant region in the previous admixture mapping analysis. eHiCA revealed more and stronger interactions between rs4761988 and *SLC4A8, SCN8A* and *FIGNL2* in EU compared to AF genome (Figure 2), consistent with the admixture mapping result. We also replicated eight known AD loci (*ABCA7, APOE, CLU, FERMT2, GRN, RASGEF1C, SORL1, TREM2*) primarily identified in EU studies, likely reflecting the high EU background of the PR population.

**Conclusion:**

Meta‐analysis in the PR population identified a novel genome‐wide significant locus. eHiCA identified several potential target genes at this novel locus, including *SCN8A*. Differential chromatin interactions between EU and AF ancestries may contribute to the significant admixture mapping signal.